# Functional and effective connectivity alterations relate to cognitive performance in aquaporin-4 antibody-positive neuromyelitis optica spectrum disorder

**DOI:** 10.1093/braincomms/fcag334

**Published:** 2026-09-01

**Authors:** Elisa Leveraro, Emilio Cipriano, Giada Lombardi, Daniela Currò, Francesco Tazza, Giovanni Novi, Alice Laroni, Paola Gazzola, Maria Malentacchi, Loredana Petrucci, Caterina Lapucci, Matilde Inglese

**Affiliations:** Department of Neuroscience, Rehabilitation, Ophthalmology, Genetics, and Mother–Child Health (DINOGMI), University of Genoa, Genoa 16132, Italy; Department of Physics (DIFI), University of Genoa, Genoa 16146, Italy; Department of Neuroscience, Rehabilitation, Ophthalmology, Genetics, and Mother–Child Health (DINOGMI), University of Genoa, Genoa 16132, Italy; Institute of Psychiatry, Psychology and Neuroscience (IoPPN), King’s College London, London SE5 8AB, UK; Department of Neurology, Ospedale San Paolo, Savona 17100, Italy; Department of Neuroscience, Rehabilitation, Ophthalmology, Genetics, and Mother–Child Health (DINOGMI), University of Genoa, Genoa 16132, Italy; IRCCS Azienda Ospedaliera Metropolitana—IRCCS AOM, Genova 16132, Italy; IRCCS Azienda Ospedaliera Metropolitana—IRCCS AOM, Genova 16132, Italy; Department of Neuroscience, Rehabilitation, Ophthalmology, Genetics, and Mother–Child Health (DINOGMI), University of Genoa, Genoa 16132, Italy; IRCCS Azienda Ospedaliera Metropolitana—IRCCS AOM, Genova 16132, Italy; Department of Neurology, PA. Micone Hospital, ASL3, Genoa 16154, Italy; Department of Neurology and CRESM, San Luigi Gonzaga University Hospital, Orbassano 10043, Italy; Department of Neurology, Hospital Sant'Andrea, La Spezia 19124, Italy; IRCCS Azienda Ospedaliera Metropolitana—IRCCS AOM, Genova 16132, Italy; Department of Neuroscience, Rehabilitation, Ophthalmology, Genetics, and Mother–Child Health (DINOGMI), University of Genoa, Genoa 16132, Italy; IRCCS Azienda Ospedaliera Metropolitana—IRCCS AOM, Genova 16132, Italy

**Keywords:** neuromyelitis optica spectrum disorder, functional connectivity, effective connectivity, default mode network, ventral attention network

## Abstract

The neural mechanisms underlying cognitive dysfunction in aquaporin-4 antibody-positive neuromyelitis optica spectrum disorder (AQP4 + NMOSD) remain unclear. Functional connectivity (FC) studies have revealed network-level alterations, but little is known about the effective interactions that support compensatory reorganization.

To address this gap, we investigated how alterations in functional and effective connectivity within large-scale networks relate to cognitive performance in AQP4 + NMOSD.

To this end, 20 AQP4 + NMOSD patients and 20 age- and sex-matched healthy controls underwent 3-T structural MRI and resting-state functional MRI. Seed-based and region-of-interest connectivity analyses focused on the default mode network and ventral attention network. Directional interactions were assessed using dynamic causal modelling (DCM). Cognitive performance was evaluated with the Brief International Cognitive Assessment for Multiple Sclerosis and the Controlled Oral Word Association Test (COWAT).

Overall, cognition was quite preserved in patients, except for reduced COWAT scores. Functional connectivity analyses revealed increased intra-network functional connectivity but decreased anterior–posterior default mode network integration. Dynamic causal modelling showed asymmetric prefrontal–posterior interactions, with stronger top-down excitatory influences from prefrontal to posterior regions.

Taken together, these findings indicate that AQP4 + NMOSD patients exhibit altered functional brain organization despite relatively preserved cognition, suggesting a pattern of large-scale network reorganization that may support cognitive performance.

## Teaser

Leveraro *et al*. report that patients with aquaporin-4 antibody-positive neuromyelitis optica spectrum disorder show altered functional brain organization despite preserved cognition. Increased connectivity within large-scale networks and stronger prefrontal influence on posterior regions accompany reduced anterior–posterior integration, suggesting network reorganization that may support cognitive performance.

## Graphical Abstract

**Graphical Abstract:**
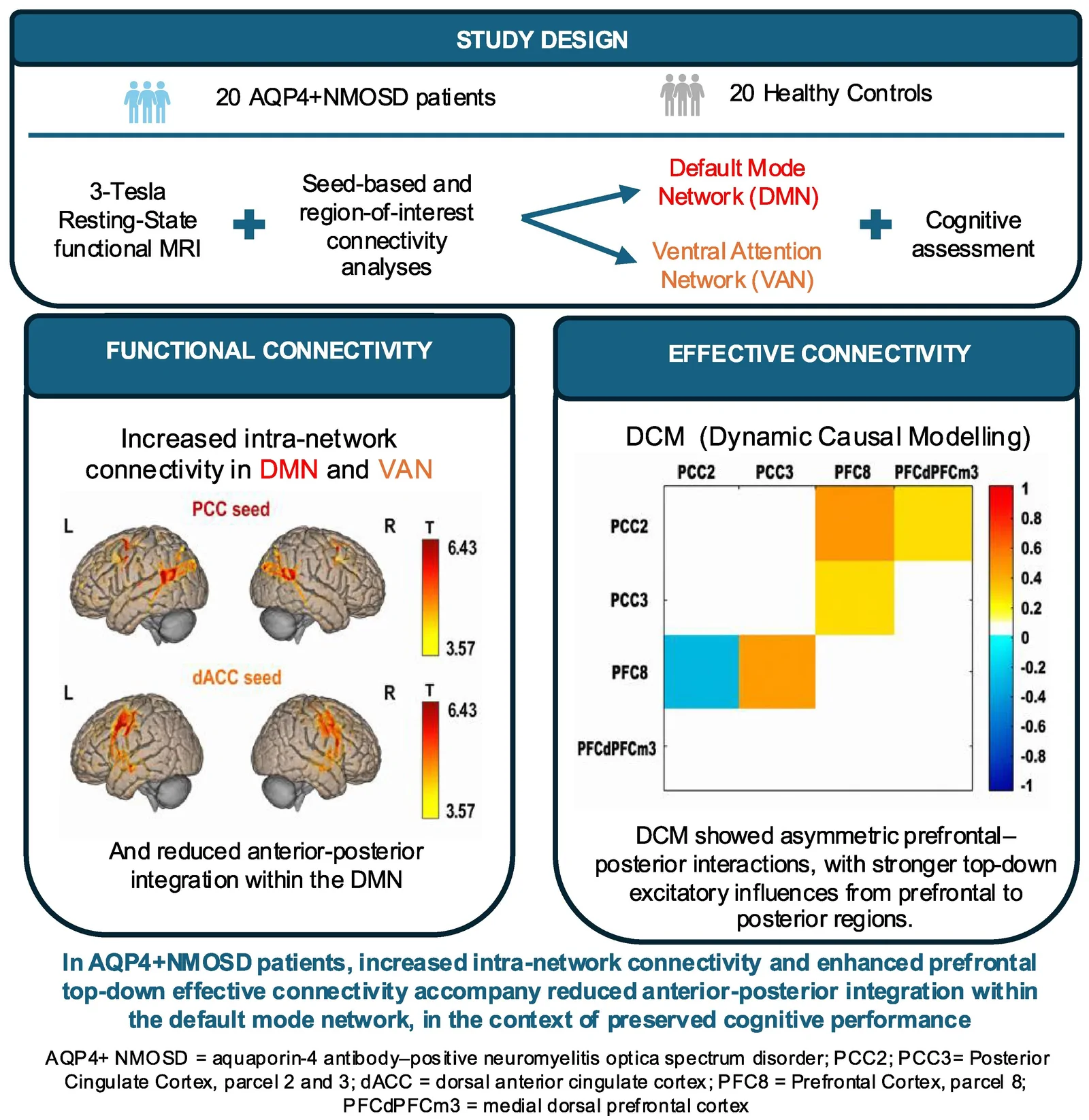

## Introduction

Neuromyelitis optica spectrum disorder (NMOSD) is a rare inflammatory disorder of the central nervous system, with most patients testing positive for specific immunoglobulin G autoantibodies that target aquaporin-4 (AQP4), a water channel protein expressed on astrocyte processes.^1-3^ Complement activation and the ensuing immune cascade lead to oligodendroglial degeneration, demyelination, and axonal damage.^4^ In recent years, the concept of sublytic astrocytopathy has gained increasing attention, suggesting that synaptopathy and synaptic loss may precede macroscopic tissue damage in patients with NMOSD.^5^ Alongside the well-known involvement of the visual and motor systems typical of NMOSD, conflicting evidence suggests cognitive involvement in these patients, with impairments observed in sustained attention, processing speed, verbal memory and executive functions.^1,6-8^ Nevertheless, the network-level mechanisms underlying these alterations and their relationship with functional reorganization remain poorly understood.^1,6-9^

Recently, resting-state functional MRI (fMRI) studies using different analytical approaches, including functional connectivity (FC), graph theoretical analysis and regional activity measures, have provided converging evidence of functional reorganization in patients with NMOSD. These studies have revealed alterations in several large-scale brain networks, including increased connectivity within the default mode network (DMN), the dorsal attention network and thalamic networks, alongside reduced connectivity within the visual and cerebellar networks.^10,11^ In addition, graph theory analyses have demonstrated reductions in both global and local network efficiency, especially in the precuneus, which is implicated in complex cognitive processes.^12,13^ Parallel studies employing amplitude of low-frequency fluctuation analyses have revealed both reduced spontaneous activity in the precuneus, posterior cingulate cortex (PCC) and lingual gyrus, as well as compensatory increases in the anterior cingulate cortex (ACC), thalamus and basal ganglia.^9,11,14^ These alterations may reflect adaptive or maladaptive mechanisms aimed at preserving cognitive efficiency in the presence of neural damage.^15^ However, their interpretation remains debated. While some authors propose compensatory functional reorganization, others view them as pathological alterations contributing to cognitive decline.^16^

Despite growing evidence of network dysfunction in NMOSD, the mechanisms underlying functional reorganization remain poorly understood. Most previous studies have relied on conventional FC analyses, which capture temporal correlations between regions but cannot determine the directionality or causal influence among network nodes. In contrast, effective connectivity (EC) approaches, such as dynamic causal modelling (DCM), provide insight into how activity in one region causally influences another, helping to distinguish adaptive compensation from impaired synaptic signalling or maladaptive network dynamics.^17^ To date, only one study has applied DCM in NMOSD, focusing on thalamocortical interactions rather than higher-order cognitive networks.^18^

Against this background, our work integrates both FC and EC analyses with cognitive testing, providing a more comprehensive characterization of large-scale network dysfunction in NMOSD. While FC captures statistical dependencies between brain regions, EC allows the investigation of directed influences within neural circuits, offering additional insights into the organization of network interactions and their relationship with cognitive performance.

To our knowledge, this is the first study to combine FC, EC and cognitive testing to investigate the relationship between network-level dysfunction and cognitive performance in NMOSD. We focused on two networks previously implicated in NMOSD and cognition: the DMN, involved in memory and executive processes, and the ventral attention network (VAN), associated with attentional control and reorienting. By comparing connectivity strength, directionality and performance correlates, we sought to determine whether network alterations reflect adaptive reorganization or disrupted synaptic signalling secondary to astrocytic injury. Given the limited sample size, an inherent challenge in monocentric studies of rare conditions such as NMOSD, these analyses were conducted within an exploratory framework and warrant replication in larger, ideally multicentric cohorts. Within this context, the present multimodal approach allows linking astrocyte-mediated synaptophysin to abnormal EC and cognitive inefficiency in NMOSD, thus advancing the understanding of network-level mechanisms underlying disease-related cognitive dysfunction.

## Materials and methods

### Participants

Twenty patients with NMOSD fulfilling the 2015 International Consensus Diagnostic Criteria and 20 age- and sex-matched healthy controls (HCs) were prospectively enrolled. Eligibility criteria for patients included seropositivity for anti-AQP4 antibodies confirmed with a commercial fixed cell-based assay, ongoing treatment with approved disease-modifying therapies for at least 12 months, absence of clinical relapses and no exposure to high-dose corticosteroids within the 4 weeks preceding MRI acquisition. For both groups, inclusion criteria were age ≥18 years and absence of contraindications to MRI. Individuals with significant ophthalmological comorbidities were excluded. The study was approved by the local ethics committee, and all procedures were conducted in accordance with the Declaration of Helsinki. Written informed consent was obtained from all participants for participation and for the use of their clinical data for research and publication purposes.

### MRI acquisition protocol

All participants underwent MRI acquisition with a 3-T MRI scanner (Prisma, Siemens Medical Systems, Erlangen, Germany) with a 64-channel head coil. The MRI acquisition protocol included: (i) three-dimensional (3D) sagittal T2-fluid attenuated inversion recovery (FLAIR) [repetition time (TR)/inversion time (TI)/echo time (TE) 5000 ms/1800 ms/393 ms; original resolution: 0.8 × 0.8 × 1 mm^3^; resolution of reconstructed images: 0.4 × 0.4 × 1 mm^3^]; (ii) 3D sagittal T1-magnetization prepared-rapid gradient echo (TR/TI/TE 2300 ms/919 ms/2.96 ms; resolution 1 × 1 × 1 mm^3^) and (iii) T2* echo-planar imaging (TR = 720 ms, TE = 33 ms, flip angle = 52°, resolution 2.3 × 2.3 × 2.3 mm^3^, time points = 600) for resting-state functional fMRI, during which participants were asked to rest with their eyes closed without falling asleep.

### Structural MRI

Firstly, lesion segmentation was performed on FLAIR and T1-weighted images using a semi-automated, user-supervised local thresholding approach (SInLAB; Siena Imaging, https://www.sienaimaging.it/, Siena, Italy). Subsequently, T1-weighted images were lesion-filled using the corresponding T1-hypointense lesion masks in the FMRIB Software Library^19^ and registered to the Montreal Neurological Institute (MNI) space through affine and non-linear transformations implemented in Advanced Normalization Tools.^20^ For all participants, brain tissue segmentation of the lesion-filled T1 images was conducted using the CAT12 toolbox within the Statistical Parametric Mapping (SPM) framework.^21,22^ Grey matter volume and white matter volume (WMV) were then extracted for each subject and normalized to the total intracranial volume.

### Functional MRI

Resting-state fMRI data were preprocessed and analysed using the CONN toolbox (v.23) implemented in MATLAB R2023b, running on the SPM framework.^23^ The preprocessing pipeline included: (i) realignment and unwarping for motion correction; (ii) slice-timing correction to account for temporal misalignment across slices; (iii) identification of potential outlier scans based on global Blood Oxygen Level Dependent (BOLD) signal and motion parameters, (iv) spatial normalization to the MNI152 standard space and (v) spatial smoothing with a Gaussian kernel of 6 mm full-width at half maximum. Following preprocessing, denoising procedures were applied to minimize the influence of motion, physiological and other artefactual sources of variance. The resulting time series were band-pass filtered within the conventional resting-state frequency range (0.01–0.08 Hz). Regions of interest (ROIs) were defined using the 200-region functional parcellation of Schaefer *et al*.^24^ in the CONN toolbox, providing a standardized and reproducible framework for network-level analyses.

### Seed-based functional connectivity analysis

Seed-based FC analysis was performed to investigate FC of the DMN and the VAN. We selected the PCC and dorsal ACC (dACC) as seeds for the DMN and the VAN, respectively, as they are commonly identified as key hubs within these networks.^11,25-27^ Seed-to-voxel connectivity maps were generated by correlating the BOLD time series of each seed with all other brain voxels.

### ROI-to-ROI functional connectivity analysis

ROI-to-ROI analysis was performed using the PCC and the dACC as reference ROIs. Mean time series from each ROI were extracted from FC maps obtained after fMRI preprocessing. For each participant, a ROI-to-ROI Pearson correlation matrix was computed and subsequently normalized using Fisher's z transformation.

### Dynamic causal modelling

DCM is an analysis framework used to model the underlying neural dynamics and direct causal interactions between brain regions, namely EC.^28^ The analyses were performed in MATLAB R2023b using the publicly available DCM/PEB code (https://github.com/pzeidman/dcm-peb-example.git) described by Zeidman *et al*.^28,29^

Differently from FC which characterizes statistical dependencies between brain regions in terms of correlation of BOLD time series, EC estimates how brain activity in one region causally affects another one.^17,28^ DCM was initially developed to estimate EC in task-based fMRI studies because it allows testing hypotheses about the directionality of information flow across brain networks and assessing how external inputs and their experimental manipulations influence the strength of interactions between nodes. To estimate intrinsic EC from resting-state fMRI data, spectral DCM (spDCM) was introduced.^30^ In spDCM, the neuronal model is defined by a simplified linear differential equation: the neural state vector (representing the activity of *N* brain regions over time) is driven by intrinsic connectivity (the A-matrix) and by random endogenous fluctuations in neural activity. Each node within the network can exert either an inhibitory (negative) or excitatory (positive) influence on other areas (between-region connectivity). In contrast, elements on the diagonal of the A-matrix represent self-connections, which are inhibitory by definition: larger positive values correspond to stronger inhibition and negative values to disinhibition.^31^ For clarity, the spDCM framework will hereafter be referred to as DCM.

Subject-level DCM was specified using a set of regions selected to represent key nodes of the networks identified in the FC analyses,^32^ thereby constraining the intrinsic A-matrix to functionally relevant pathways and reducing model dimensionality. Group-level inference on A-matrix parameters was performed within the parametric empirical Bayes (PEB) framework. The model space, defined in a hypothesis-driven manner based on the ROI-to-ROI results, was explored through Bayesian model comparison and reduction, and final parameter estimates were obtained via Bayesian model averaging. Connections with a posterior probability exceeding 0.95 (PP > 0.95) were considered significant, indicating strong evidence according to the free-energy approximation.^33-35^

### Cognitive assessment

All participants completed the Italian version of the Brief International Cognitive Assessment for Multiple Sclerosis (BICAMS)^36^ comprising the oral Symbol Digit Modalities Test (SDMT), the California Verbal Learning Test-Second Edition (CVLT-II) and the Brief Visuospatial Memory Test-Revised (BVMT-R), along with the Controlled Oral Word Association Test (COWAT).^37^ The *z*-scores were computed for all tests based on normative data corrected for age, sex and education, thereby allowing demographically adjusted group comparisons. The impairment on each BICAMS subtest was defined based on Italian normative data^36^ (*T*-score ≤ 35).

### Statistical analysis

Statistical analyses were performed using Jamovi (version 2.7.31). Group comparisons between AQP4 + NMOSD patients and HCs were performed using independent-sample *t*-tests or Mann–Whitney U tests, as appropriate. Sex distribution was compared using χ^2^ tests. Group differences in FC between AQP4 + NMOSD patients and HCs were assessed using general linear models (GLMs), corrected for education. For seed-based analyses, statistical testing was performed in the CONN toolbox using advanced family-wise error (FWE) control settings, with a cluster-level threshold of *P* < 0.05 FWE-corrected. For ROI-to-ROI analyses, connections showing significant group differences were identified using the same GLM framework, with a threshold of *P* < 0.05 FWE-corrected. Pearson correlations were performed within the AQP4 + NMOSD patient group to examine associations between z-scored cognitive test scores and mean FC of DMN and VAN, as well as significant ROI-to-ROI connectivity. Statistical significance was set at *P* < 0.05 (two-tailed). Given the limited sample size, no correction for multiple comparisons was applied in order to reduce the risk of Type II errors; accordingly, these analyses should be considered exploratory.

## Results

### Demographics, brain volumes and cognitive performance

**Figure 1:**
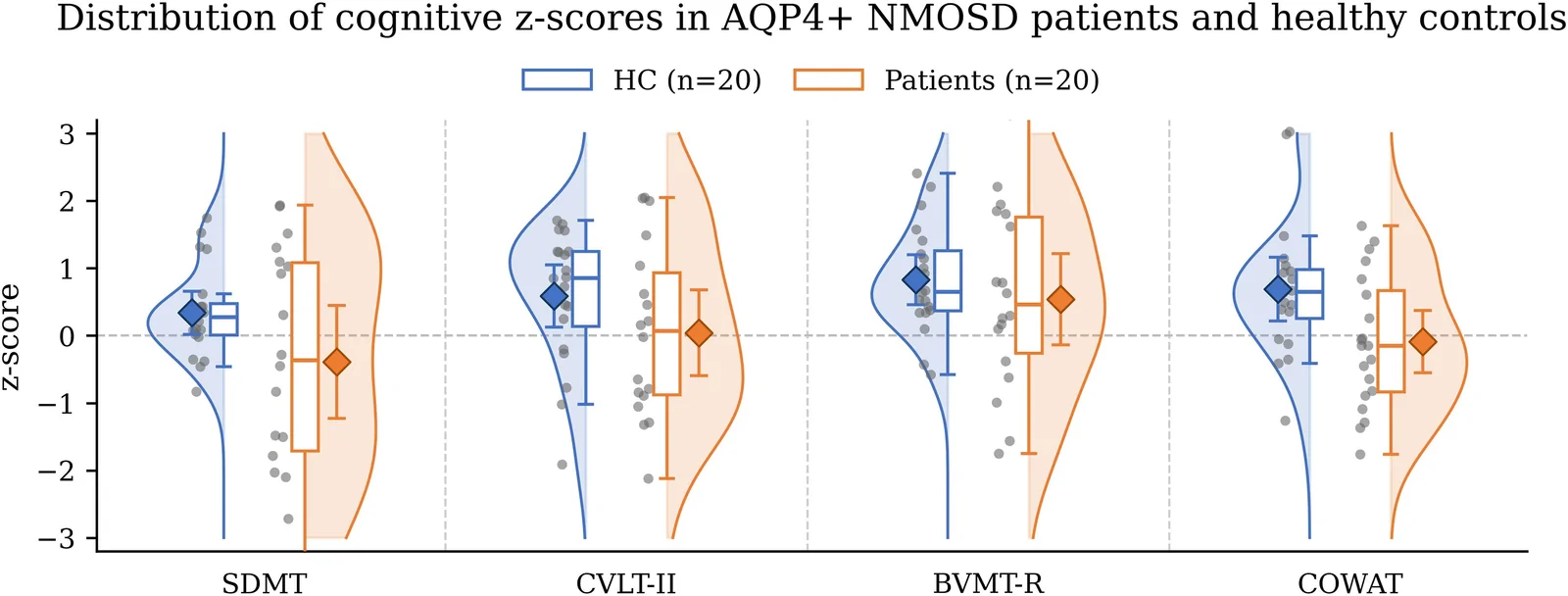
Distribution of cognitive *z*-scores in AQP4 + NMOSD patients and HCs. Half-violin plots illustrate the distribution density of *z*-scores for each cognitive measure, while overlaid boxplots represent the median and inter-quartile range. Individual data points correspond to single participants. Diamonds indicate group means, with error bars representing the standard error of the mean. HCs (*n* = 20) are shown in blue, and patients with aquaporin-4 antibody-positive neuromyelitis optica spectrum disorder (AQP4 + NMOSD, *n* = 20) are shown in orange. HC, healthy controls; AQP4 + NMOSD, aquaporin-4 antibody-positive neuromyelitis optica spectrum disorder; SDMT, Symbol Digit Modalities Test; CVLT-II, California Verbal Learning Test-Second Edition; BVMT-R, Brief Visuospatial Memory Test-Revised; COWAT, Controlled Oral Word Association Test.

AQP4 + NMOSD patients had fewer years of education compared HCs, whereas age and sex distributions did not differ between groups (Table 1). Total normalized WMV was significantly lower in patients than in HCs (Table 1). Overall cognitive performance was comparable between groups, except for COWAT, where AQP4 + NMOSD patients showed lower *z*-scores relative to HCs (Table 1; Fig. 1).

**Table 1:** Summary of demographic, clinical and MRI data

	AQP4 + NMOSD	HCs	*P*-value
Sample size, *n*	20	20	
Age (years), mean (SD)	53.53 (±14.14)	52.2 (±12.64)	0.44
Education (years), mean (SD)	12.15(±2.56)	16.6 (±2.46)	**<0.001**
Sex (females/males)	18/2	14/6	0.11
DD (years), mean (SD)	9.15 (±5.2)		
EDSS, median (range)	3.27 (0–6)		
DMTs			
Rituximab, *n*	14		
Ravulizumab, *n*	2		
Satralizumab, *n*	2		
Eculizumab, *n*	2		
MRI metrics			
Lesion volume (ml), mean (SD)	1.65 (±2.71)		
nGMV (ml), mean (SD)	0.44 (±0.03)	0.44 (±0.02)	0.83
nWMV (ml), mean (SD)	0.33 (±0.022)	0.35 (±0.02)	**0.017**
Cognitive performance			
SDMT, *z*-score, mean (SD)	−0.39 (±1.64)	0.34 (±0.68)	0.24^a^
Impaired on SDMT, *n* (%)	6 (30%)		
CVLT-II, *z*-score, mean (SD)	0.042 (±1.24)	0.59 (±0.98)	0.15
Impaired on CVLT-II, *n* (%)	1 (5%)		
BVMT-R, *z*-score, mean (SD)	0.54 (±1.33)	0.83 (±0.79)	0.42^a^
Impaired on BVMT-R, *n* (%)	2 (10%)		
COWAT, *z*-score, mean (SD)	−0.09 (±0.99)	0.69 (±1.02)	**0.017**
Impaired on COWAT, *n* (%)	1 (5%)		
Impaired on ≥1 subtest, *n* (%)	7 (35%)		
Impaired on ≥2 subtests, *n* (%)	3 (15%)		

Clinical and demographic characteristics of the study sample. Significant differences between patients and HCs are indicated in bold. HCs, healthy controls; AQP4 + NMOSD, patients with aquaporin-4 antibody-positive neuromyelitis optica spectrum disorder; SD, standard deviation; DD, disease duration; EDSS, Expanded Disability Status Scale; DMTs, disease-modifying therapies; nGMV, normalized grey matter volume; nWMV, normalized white matter volume; SDMT, Symbol Digit Modalities Test; CVLT-II, California Verbal Learning Test-Second Edition; BVMT-R, Brief Visuospatial Memory Test-Revised; COWAT, Controlled Oral Word Association Test.

^a^Mann–Whitney U test applied; Student's *t*-test used for all other comparisons.

### Seed-to-voxel functional connectivity

**Figure 2:**
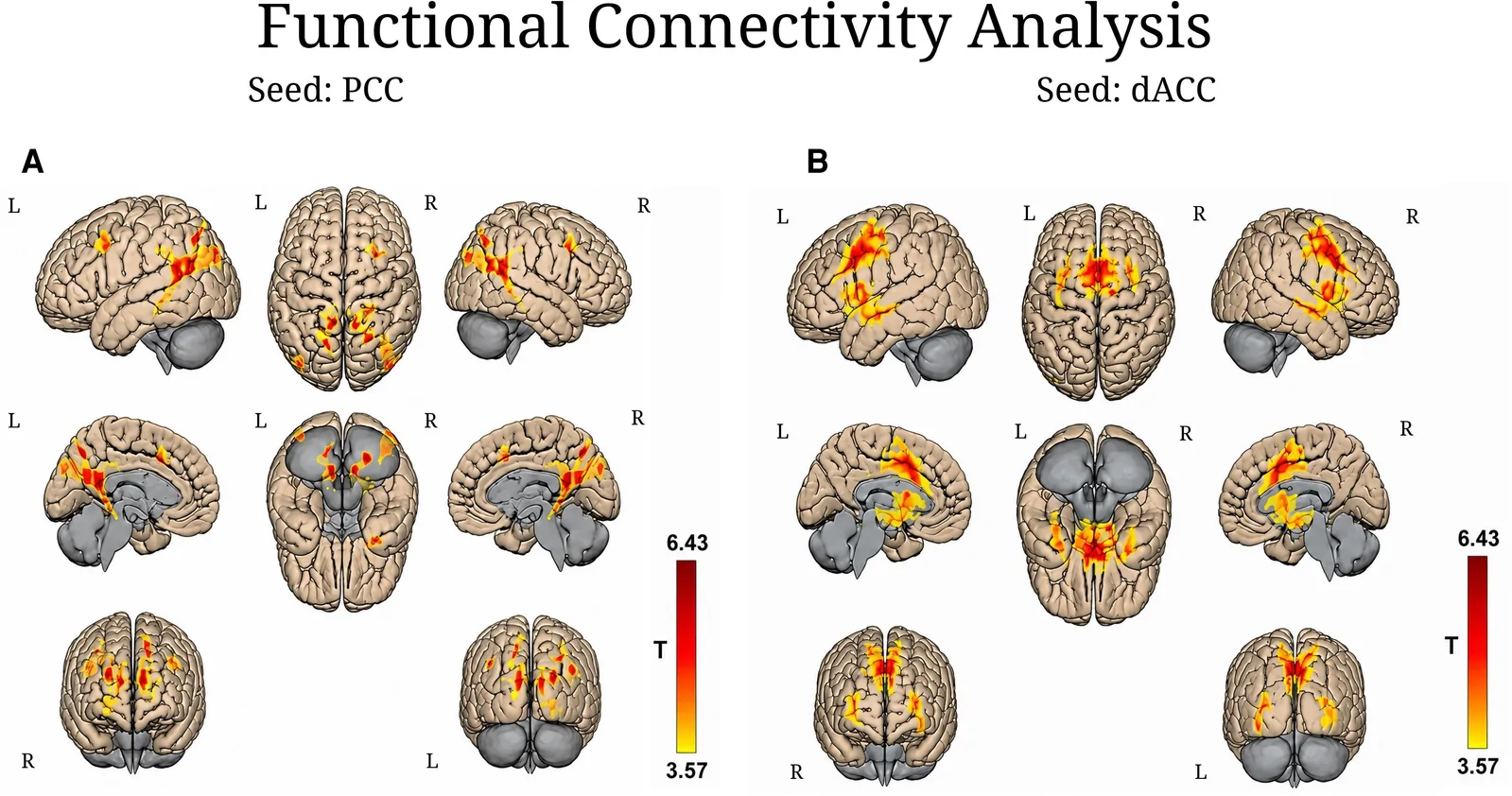
Seed-based FC in AQP4 + NMOSD patients versus HCs. (**A**) DMN, selected seed: PCC; (**B**) VAN, selected seed: dACC. FC was compared between AQP4 + NMOSD patients (*n* = 20) and HCs (*n* = 20) using GLMs. Significant clusters correspond to NMOSD > HC (pFWE < 0.05, cluster size > 100 voxels). Higher colour intensity indicates greater *T*-values (*T*) and therefore larger functional connectivity differences between groups. AQP4 + NMOSD, aquaporin-4 antibody-positive neuromyelitis optica spectrum disorder.

Compared with HCs, AQP4 + NMOSD patients showed (i) increased FC between the PCC seed and posterior cortical regions, including the lateral occipital cortices, precuneus, superior parietal cortex and right middle frontal gyrus (Fig. 2A); (ii) increased FC between the dACC seed and bilateral supplementary motor areas, paracingulate gyri, superior frontal gyri, insula and putamen (Fig. 2B). Cluster coordinates, sizes and peak regions are reported in Supplementary Table 1.

### ROI-to-ROI functional connectivity

ROI-to-ROI analysis revealed reduced FC within the DMN. Specifically, decreased FC was observed between the right PCC and the left dorsolateral prefrontal cortex (PCC2: *T*(38) = −3.40, false discovery rate (FDR)-corrected p-value (pFDR) = 0.025, *d* = 1.10; PCC3: *T*(38) = −4.39, pFDR = 0.002, *d* = 1.42), as well as between the right PCC and the right medial prefrontal cortex (PCC2: *T*(38) = −4.55, pFDR = 0.006, *d* = 1.48; PCC3: *T*(38) = −4.53, pFDR = 0.006, *d* = 1.47).

### Dynamic causal modelling

**Figure 3:**
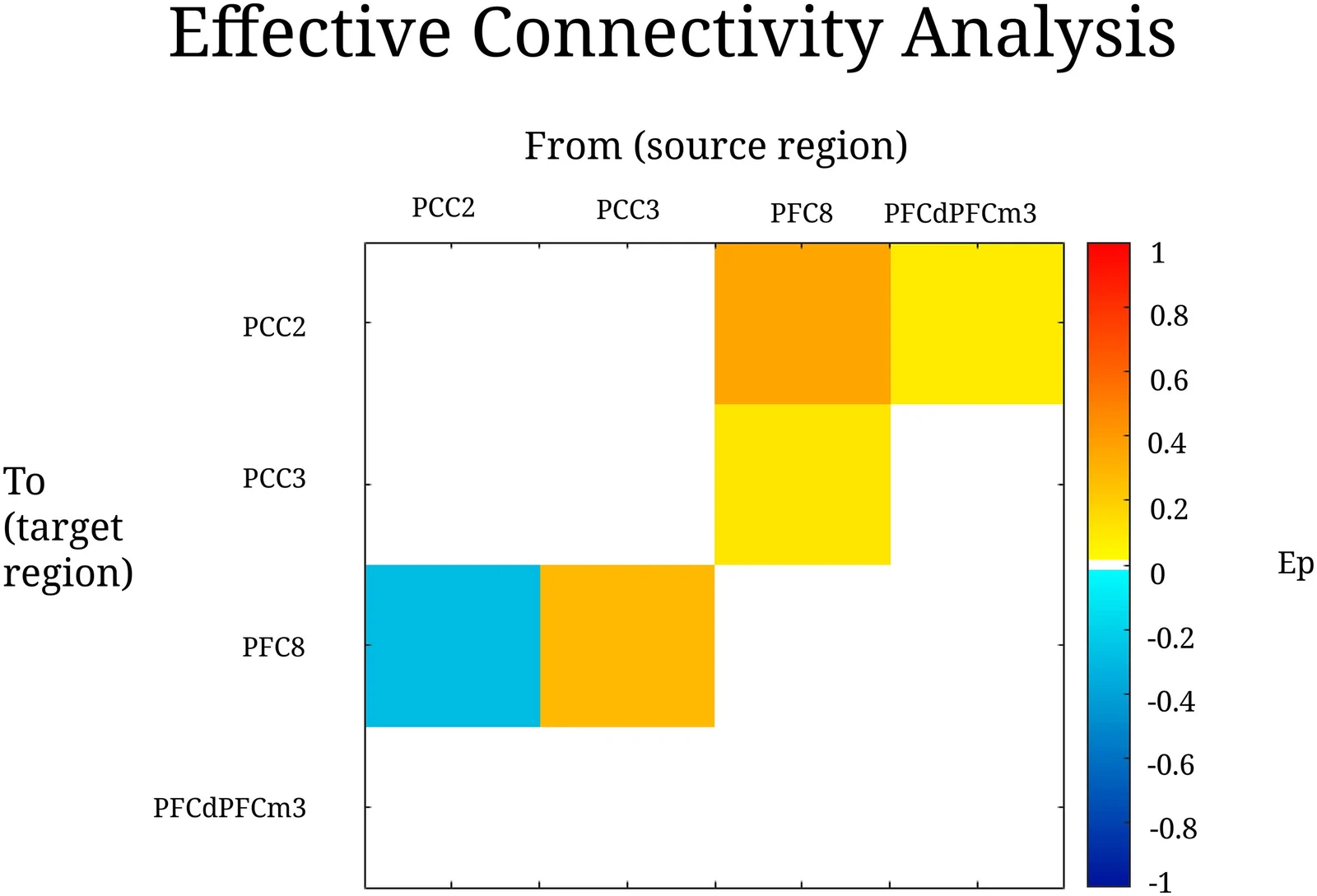
Results of the DCM analysis represented as EC matrices. EC parameters were estimated using PEB. Only connections showing strong evidence at the group level (PP > 0.95) are displayed. Connectivity strength (Ep) is depicted on a colour scale ranging from blue (inhibitory) to red (excitatory). Off-diagonal parameters represent between-region coupling in Hz. Each observation corresponds to one participant (AQP4 + NMOSD, *n* = 20). AQP4 + NMOSD, aquaporin-4 antibody-positive neuromyelitis optica spectrum disorder; PCC2, posterior cingulate cortex, parcel 2; PCC3, posterior cingulate cortex, parcel 3; PFC8, prefrontal cortex, parcel 8; PFCdPFCm3, medial dorsal prefrontal cortex, parcel 3.

PEB analysis of intrinsic (A-matrix) parameters revealed significant directed interactions among DMN nodes. Specifically, connectivity estimates were as follows: PCC2 → PFC8 = −0.29 Hz (inhibitory), PCC3 → PFC8 = 0.28 Hz (excitatory), PFC8 → PCC3 = 0.11 Hz (excitatory), PFC8 → PCC2 = 0.35 Hz (excitatory) and PFCdPFCm3 → PCC2 = 0.08 Hz (excitatory) (Fig. 3). These results indicate asymmetric reciprocal interactions between PCC and prefrontal cortex regions, with PFC8 exerting the strongest excitatory influence on PCC2, whereas PCC2 exerts an inhibitory effect on PFC8. The excitatory drive from PCC3 to PFC8 was notably stronger than the reverse influence, and the PFCdPFCm3 → PCC2 connection was positive but modest in magnitude.

### Associations between functional connectivity and cognitive performance

Mean DMN connectivity was positively correlated with visuospatial memory (*r* = 0.56, *P* = 0.014) and verbal fluency (*r* = 0.46, *P* = 0.038). Higher SDMT performance was associated with stronger connectivity between the posterior cingulate–precuneus cluster and both the left dorsolateral (*r* = 0.60, *P* = 0.008) and right medial prefrontal cortex (*r* = 0.52, *P* = 0.025). COWAT *z*-scores also correlated with connectivity between the posterior cingulate–precuneus cluster and the medial prefrontal cortex (*r* = 0.53, *P* = 0.014).

## Discussion

In the present study, AQP4 + NMOSD patients showed largely preserved cognitive performance despite reduced WMV, with impairment limited to verbal fluency. Functional MRI analyses revealed increased connectivity within the DMN and VAN, reduced anterior–posterior DMN integration and altered patterns of EC characterized by enhanced prefrontal influence on posterior regions. Together, these findings suggest the presence of large-scale network reorganization in the context of relatively preserved cognition. This dissociation between structural brain alterations and maintained cognition may be consistent with a pattern of large-scale functional reorganization within distributed networks. Consistent with this interpretation, connectivity–behaviour associations indicated that stronger mean DMN connectivity related to better visuospatial memory and verbal fluency, while increased FC between PCC and prefrontal regions was associated with higher SDMT and fluency performance. Importantly, these associations were of moderate magnitude, supporting a contributory role of network-level integration in cognitive efficiency. In rare conditions such as AQP4 + NMOSD, where large samples comparable to those available in multiple sclerosis (MS) are inherently difficult to obtain, these findings should be interpreted within the constraints of cohort size. However, given the exploratory nature of these analyses, larger studies are needed to confirm these relationships. Additionally, early initiation of high-efficacy immunosuppressive therapies may have contributed to preserved cognitive performance in our cohort, potentially acting as a moderating factor on both structural damage and functional network organization. In support of this hypothesis, recent evidence has shown lower rates of subcortical grey matter atrophy in AQP4 + NMOSD patients who initiated treatment early.^38^ This may partly account for discrepancies with previous studies reporting higher rates of cognitive impairment in AQP4 + NMOSD.^7,8,39^ Future longitudinal studies integrating cognitive assessments, advanced MRI measures and biomarkers of synaptic dysfunction may help clarify whether early immunotherapy influences not only relapse prevention and overt disability but also more subtle manifestations of the disease, including cognitive dysfunction. Seed-to-voxel analyses revealed increased FC within the DMN, particularly between the PCC and both posterior and frontal regions, along with hyperconnectivity of the dACC with anterior and insular areas. Such widespread synchronization may reflect a reconfiguration of large-scale network dynamics, potentially supporting more efficient interregional communication in the presence of subtle or diffuse structural alterations. A broader interpretation of these findings can be further contextualized through comparison with other neurological conditions characterized by network-level alterations. In MS, increased FC, particularly within the DMN and frontoparietal networks, has frequently been reported in patients with preserved cognition and interpreted as a potential adaptive response, although this pattern may evolve towards network inefficiency with disease progression.^10,40-42^ Within this broader framework, the pattern observed in AQP4 + NMOSD, characterized by increased connectivity alongside preserved cognitive performance, may reflect a form of large-scale functional reorganization that is not disease-specific but instead represents a generalizable network response to brain pathology. This distributed synchronization may therefore index an adaptive attempt to sustain cognitive performance by enhancing interregional communication despite underlying structural compromise.

Conversely, ROI-to-ROI analyses demonstrated reduced functional integration between anterior and posterior DMN areas, specifically between the PCC and medial/dorsolateral prefrontal cortex, suggesting disrupted long-range coherence accompanied by increased local cohesion in posterior regions. This pattern may represent a ‘maladaptive rebalancing’, a phenomenon observed in other inflammatory and degenerative conditions, where network activity becomes less efficiently directed as secondary pathways are strengthened at the expense of global network efficiency.^15,43^

EC analysis using DCM provided critical insights into the directionality of these interactions and directed network reorganization in this population. The observed pattern indicates that prefrontal regions exert stronger excitatory influences on the PCC, while the PCC provides inhibitory feedback to prefrontal nodes, an asymmetric top-down pattern consistent with compensatory prefrontal control aimed at sustaining network synchronization and mitigating intrinsic disconnection.^44^ Taken together, these findings suggest that preserved cognition in AQP4 + NMOSD patients may depend on dynamic, compensatory reorganization within large-scale brain networks. Despite the presence of structural alterations and connectivity changes, patients exhibit globally preserved cognitive performance. This suggests that the brain network may engage functional compensatory mechanisms, whereby the prefrontal cortex assumes a more active role in coordinating the DMN, thereby compensating for reductions in global network efficiency.

### Limitations

Some limitations must be acknowledged. First, the relatively small sample size and cross-sectional design preclude causal inferences about the temporal evolution of the observed connectivity alterations and their relationship with cognitive outcomes. Future longitudinal studies will be important to increase statistical power, improve generalizability and clarify whether the observed connectivity changes reflect adaptive, maladaptive or disease-related processes over time. In addition, although patients and HCs were matched for sex, the limited sample size did not allow adequately powered sex-stratified analyses. Larger cohorts will be needed to further investigate the potential influence of sex on functional and EC alterations in AQP4 + NMOSD.

Second, the potential impact of immunosuppressive therapies on FC cannot be excluded, as these agents may modulate neuroinflammation and neural signalling. Furthermore, our findings are limited to cognitively preserved AQP4+ patients and may not generalize to individuals with more severe cognitive impairment.

Finally, visual function was not systematically assessed in the present study. Although participants with significant ophthalmological comorbidities were excluded, a potential influence of subclinical or residual visual impairment on performance in visually demanding cognitive tasks cannot be entirely excluded.

## Conclusion

In summary, our results indicate that in AQP4 + NMOSD patients exhibit altered functional brain organization in the context of relatively modest structural changes and largely preserved cognitive performance. While seed-based analyses revealed hyperconnectivity, ROI-to-ROI analyses uncovered disrupted anterior–posterior integration, and DCM revealed altered EC characterized by increased prefrontal influence over posterior regions. Together, these findings are consistent with a pattern of large-scale network reorganization that may support preserved cognition. By combining volumetric, functional and causal modelling approaches, this study provides novel insights into DMN dynamics in NMOSD and highlights potential neural substrates of cognitive reserve in this disorder.

## Supplementary Material

fcag334_Supplementary_Data

## Data Availability

The data presented in this study are available upon request from the corresponding author.
