# Supplementary material for: Functional and effective connectivity alterations relate to cognitive performance in aquaporin-4 antibody-positive neuromyelitis optica spectrum disorder
Source: Brain Commun. 2026 Sep 1;8(5):fcag334. doi: 10.1093/braincomms/fcag334 (PMC13563299; doi:10.1093/braincomms/fcag334)
Supplement: Supplementary file 1 [file fcag334_supplementary_data.docx]

**Supplementary Table 1:** Seed-based functional connectivity clusters (pFWE < 0.05)

| **Seed** | **Cluster** | **Peak coordinates (x, y, z)** | **k (voxels)** | **Main regions included** |
| --- | --- | --- | --- | --- |
| PCC | 1 | +22, −62, +26 | 383 | Right supracalcarine cortex, right occipital cortex |
|  | 2 | −10, −50, +16 | 363 | Precuneus |
|  | 3 | +42, −84, +28 | 288 | Right superior lateral occipital cortex |
|  | 4 | +28, +12, +38 | 108 | Right middle frontal gyrus |
|  | 5 | +24, −36, +42 | 108 | Superior parietal cortex |
|  | 6 | −38, −82, +34 | 106 | Left superior lateral occipital cortex |
| dACC | 1 | −4, +12, +38 | 1783 | SMA, paracingulate gyrus |
|  | 2 | −32, +4, +4 | 419 | Left SMA, insula, putamen |
|  | 3 | +30, +8, +14 | 281 | Right SMA, paracingulate gyrus, superior frontal gyrus |
| **Supplementary Table 1:** Cluster coordinates, sizes, and peak regions for each seed-based functional connectivity clusters. The Harvard-Oxford atlas was used to identify the brain regions corresponding to the clusters that showed statistical differences between the groups.  **Abbreviations**: PCC: posterior cingulate cortex; dACC: dorsal anterior cingulate cortex: SMA: Supplementary Motor Area. | | | | |
